# Relevance of Adherence Monitoring in Adult Patients With Growth Hormone Deficiency Under Replacement Therapy: Preliminary Monocentric Data With Easypod^TM^ Connect

**DOI:** 10.3389/fendo.2019.00416

**Published:** 2019-06-28

**Authors:** Antonio Mancini, Edoardo Vergani, Carmine Bruno, Andrea Palladino, Alessandro Brunetti

**Affiliations:** Operative Unit of Endocrinology, Fondazione Policlinico Universitario A. Gemelli IRCCS, Catholic University of the Sacred Heart, Rome, Italy

**Keywords:** growth hormone, Easypod^TM^, compliance, IGF-1, personalized therapy

## Abstract

**Purpose:** Non-compliance to recombinant human growth hormone (r-hGH) therapy in adult growth hormone deficiency (aGHD) is a major concern for endocrinologist, as it affects significantly efficacy outcomes. This 12-month observational study was aimed to assess adherence in GHD patients treated with r-hGH administered via Easypod^TM^, an electronic, fully automated injection device designed to track the time, date and dose administered.

**Methods:** 65 patients receiving r-hGH therapy were included in the study and 32 completed the study. The primary endpoint, adherence to treatment, was calculated as the proportion of injections correctly administered during the observational period out of the expected total number of injections. Adherence, tracked by the Easypod^TM^, was evaluated at months 6 (V1) and 12 (V2) after baseline (V0). As secondary end-point, serum IGF-1 levels were also determined.

**Results:** The Easypod^TM^ data showed a median adherence of 80% throughout the period V0-V2. Females are more compliant than males. Adherence levels are correlated to IGF-1 ones.

**Conclusions:** Adherence is connected with therapy efficacy in aGHD. The injection-recording system and other characteristics of Easypod^TM^ could enhance the ability of physicians to monitor adherence to r-hGH treatment, identifying non-compliant patients, thus enabling physicians to modify their management to maximize the benefits of the treatment.

## Introduction

Adult growth hormone deficiency (aGHD) is a disorder characterized by a reduction in GH secretion caused by congenital or acquired diseases affecting the hypothalamus or pituitary gland (1). The consequences of GHD are several, including: reduced quality of life, depressed mood, decreased bone density, increased risk of developing non-alcoholic fatty liver disease (NAFLD) and increased cardiovascular risk, due to changes in cardiac mass and performance, visceral obesity, insulin resistance, dyslipidemia, vascular atherosclerosis, endothelial dysfunction, hypercoagulability and increased secretion of pro-inflammatory cytokines (2, 3).

This clinical entity can be treated by replacement therapy with recombinant human growth hormone (r-hGH) (4). Several studies and meta-analyses have shown a positive effect of r-hGH treatment on metabolic dyscrasia, cardiovascular morbidity, and fracture risk, showing reassuring data on malignancy risk and regrowth/recurrences during long treatment protocols (5).

A major cause of failure of r-hGH therapy can be patients' non-compliance and non-persistence with the prescribed regimen. Compliance has been defined as the extent to which a patient's behavior coincides with the clinical prescription or with medical advice (6) or, more concretely, the extent to which patients take medication according to the regimen prescribed by their health-care providers (7).

Persistence has been considered as the percentage of patients continuing to use therapy after a specific period, based on pharmacy prescription refill records (8). Poor compliance with this therapy is a common finding and it affects efficacy outcomes and increases healthcare costs (9). Rosenfeld et al estimated that non-compliance with GH therapy in adults is around 65% (6). Misperceptions about the consequences of missed GH doses, discomfort with injections, dissatisfaction with treatment results (especially in aGHD where there is no linear growth after the treatment), and inadequate contact with health-care providers were strongly associated with non-compliance (6).

Another relevant problem is that patients' compliance may be evaluated directly only using patients' self-reports, with a significant risk of overestimation of the reported rates, while the most used undirected strategy to trace patients compliance, and consequently to titrate r-hGH doses, is insulin-like growth factor 1 (IGF-1) variation during the follow-up (10). Although IGF-1 increase, according to age and sex specific IGF-1 SDS, indicates adequate therapy, it does not allow to quantify patients' adherence levels objectively.

Recently, new devices for r-hGH administration have made their appearance, including syringes with needle, injection pens, self-injection pens, needle-free devices and electronic devices (11). As reported by Dumas et al. (12), an optimal r-hGH device should respond to the following features: reliability, ease of use, lack of pain during injection, safety in use and storage and minimum number of steps before injection preparation. Furthermore, the objective monitoring of adherence, via a good tracking system could be a guarantee of adequate treatment for physicians (13). The Easypod^TM^ device is the only electronic GH injection device available and it was developed to give unbiased real-time data on patients' adherence (13). Thanks to its skin sensor it ensures drug intake. Patients share their data at intervals and their healthcare providers can monitor at any time, on a secure web-based platform, progress in the therapy (14).

Thus, the aim of this monocentric, observational and prospective study is to analyze the data given by Easypod^TM^, during a 1-year follow-up, to trace aGHD patients' adherence to r-hGH therapy, defining the importance of an adequate compliance in such a sneaky disease.

## Materials and Methods

This was an observational, prospective study with the primary aim of monitoring the rate of adherence to r-hGH treatment for 1 year in adult patients affected by GHD. The enrolled patients received r-hGH therapy (1–1.5 mg per week) with the Easypod^TM^ Clinical Kit, a system comprised of an electronic automated injection device (Easypod^TM^) with a docking station for recording r-hGH administration data to enable objective monitoring of actual drug usage. The secondary end-point was to monitor the effect of r-hGH treatment on serum IGF-1.

Sixty five subjects involved in this study (35 females, 30 males) were admitted to the “Policlinico Gemelli” University Hospital Dept. of Internal Medicine and were enrolled after being given an explanation of purposes and nature of the study, conducted in accordance with the declaration of Helsinki, as revised in 2013. The study protocol was approved by the Institutional review board of “Medical Pathology” of our Hospital.

GHD was diagnosed with dynamic test, using Growth Hormone-Releasing Hormone (GHRH 50 ug i.v. + arginine 0,5 g/kg), with a peak GH response < 9 μg/L when BMI was < 30 kg/mq or < 4 μg/L when BMI was > 30 kg/mq. The test was performed in patients with known pathologies of the hypothalamic-pituitary axis, or affected by clinical signs such as troncular obesity, osteoporosis and fatigue and hormonal alterations such as IGF-1 deficiency. A Magnetic Resonance Imaging (MRI) was performed to establish the etiology of hormone deficiency; patients with normal cerebral appearance were diagnosed idiopathic GHD. The test was performed after an adequate replacement therapy of other pituitary-dependent axes; in particular, 22 patients were under levothyroxine treatment for secondary hypothyroidism, 21 patients under hydrocortisone treatment for secondary hypoadrenalism, 16 patients under gonadal steroids (14 males under testosterone undecanoate and 2 females under estro-progestinic therapy) for secondary hypogonadism; finally, 2 patients assuming desmopressin for insipidus diabetes. All therapies were monitored by measuring peripheral hormone levels as appropriate.

Only patients over 18 years, with a diagnosis of GHD according to the previous criteria, under r-hGH (Saizen®, Merck KGaA, Darmstadt, Germany) treatment, equipped with Easypod^TM^ and after giving a written consent were included.

Exclusion criteria were corticosteroid treatment (except for topic, inhalatory, and oral hydrocortisone as replacement regimen), malnutrition, malabsorption, bone dysplasia, severe liver dysfunction, active malignancy, history of cranial hypertension or active cranial hypertension, decompensated type 1 or 2 diabetes mellitus and autoimmune diseases under immunosuppressive treatment. Patients could decide to discontinue their participation at any time.

Following these requirements and excluding patients who did not complete the observational period, 32 patients, aged 24–72 years (mean 54.1), 19 females, and 13 males completed the follow-up. This group included 7 naive patients and 25 patients who were on rhGH treatment (previously started, with a range of 6–24 months treatment), tested without a wash-out period. The r-hGH dose was stable along the whole follow up period in all patients (and for at least 6 months before inclusion for non-naive patients).

As regards the other 33 patients, only in 4 patients r-hGH was prudentially discontinued for clinical reasons, even if not clearly related to r-hGH (suspicion of sleep apnoea syndrome in 1 patient, monoclonal gammopathy of uncertain significance in 1 patient, lymphoproliferative disorder in 1 patient, thrombocytopenia in 1 patient); in the others for personal motivations (inaccurate respect of scheduled controls, travels or other familiar problems, difficulties in r-hGH supply). In 6 subjects drop-out occurred within the first semester of treatment, the remaining ones were not considered in the results for the reasons above mentioned, which prevented an objective assessment of compliance in the follow-up period.

**Figure 1:**
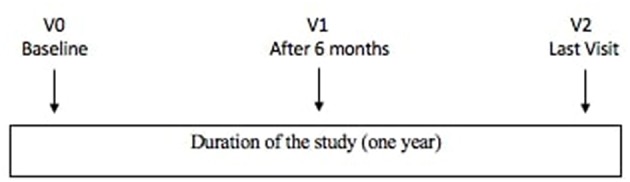
Study design.

The study design is represented in Figure 1.

The study duration for each recruited patient was 1 year.

Each patient was enrolled in the study at baseline visit, after the assessment of eligibility criteria. Easypod^TM^ devices were supplied by Merck Serono SpA (as usual for Saizen therapy). A support service, provided as part of Merck Serono SpA Patient Support Program, was guaranteed to the enrolled patients in order to train them in correct device usage and replacement procedures, or management of malfunction of the device occurring during the study.

Pursuant to the observational nature of the study, the r-hGH treatment was administered according to routine clinical practice, independent of patients' participation in the study. The outcome measurements for both the primary and the secondary endpoints were assessed at visits V0, V1, and V2, respectively. The adherence to the treatment for each patient was derived from injections recorded in the device and it was estimated as the proportion of injections correctly administered during the observational period out of the expected total number of injections. The target rate for adherence was set as ≥80%, while full adherence was defined as ≥92% at the start of the study.

Blood samples were collected after overnight fasting into pyrogen-free tubes with heparin as anticoagulant; in all patients the following parameters were determined:

– basal determination of IGF-1
– basal determinations of metabolic parameters: glucose, total cholesterol, low-density lipoprotein (LDL), high-density lipoprotein (HDL) and triglycerides.

Plasma concentrations of glucose, total cholesterol, HDL-cholesterol, triglycerides, uric acid, albumin were measured by using enzymatic assays and on Olympus AU2700 chemistry analyzer (Olympus America Inc., Center Valley, PA, USA). The intra-and inter-assay coefficients of variation (CV) for total cholesterol and triglycerides were <1.5 and <2.5%, respectively. The intra-and inter-assay CV for HDL-cholesterol were <2.5 and <3.0%, respectively. LDL cholesterol was calculated by Friedewald's equation: LDL = total cholesterol–(HDL + triglycerides/5).

Serum concentrations of IGF-1 were measured by using immunochemiluminometric assays on a Roche Modular E170 analyzer (Roche Diagnostics, Indianapolis, IN, USA). The intra- and inter-assay CV for all hormones were, respectively, <5.0 and <7.0%.

The statistical analysis was performed using Stata 13. The modifications of hormonal and metabolic parameters between the visits were evaluated by *t*-test for paired data. The comparison between IGF-1 values in groups composed of males and females was performed using the Mann-Whitney test, due to the small number of subjects in the two groups. Linear regression and Spearman correlation coefficient were used to investigate the association between adherence and IGF-1 variation throughout the follow up. A value of *P* <0.05 was considered statistically significant.

## Results

A total of 65 patients (35 females and 30 males) were enrolled in this study, of whom only 32 (19 females and 13 males, 25 non-naive and 7 naive to GH-treatment at inclusion) were considered in the results. The causes of aGHD in our population were distributed as follows: 31% idiopathic, 31% empty sella, 16% post-surgical hypopituitarism, 10% non-productive adenomas, 6% Sheehan's syndrome, and 6% childhood onset GHD. Mean ± SEM IGF-1 levels at the time of diagnosis were 121.9 ± 11.4 ng/ml; Mean ± SEM GH peak after GHRH+arginine administration was 4.1 ± 0.6 μg/L in patients with BMI <30 kg/m^2^ and 2.7 ± 0.4 μg/L in patients with BMI≥30 kg/m^2^.

Table 1 shows median adherence rates in our population and the difference between male and female population.

**Table 1:** Median adherence rate during a 1-year follow up.

**Follow up**	**Population**	**Median adherence rates**
Between V0 and V1	Whole Population	85%
	Females	87%
	Males	76%
Between V1 and V2	Whole Population	79%
	Females	81%
	Males	73%
Between V0 and V2	Whole Population	80%
	Females	84%
	Males	71%

No significant differences between mean ± SEM body mass index (BMI), triglycerides, LDL, HDL, measured at V0, V1, and V2 were detected in the whole population (Table 2) and in the groups divided according to sex (Table 3), although a trend toward lower triglycerides and higher HDL levels was observed in the female subgroup. There were no significant differences in serum glucose either.

**Table 2:** Mean ± SEM serum metabolic parameters and BMI in V0, V1, V2 in the whole population.

	**V0**		**V1**		**V2**	
	**Mean**	**SEM**	**Mean**	**SEM**	**Mean**	**SEM**
Blood glucose (mg/dl)	83	2.4	84	3.4	84.7	2.5
Triglycerides (mg/dl)	159.65	18.66	144.6	15	141.5	14.5
HDL (mg/dl)	44.43	3.13	49.31	4.02	52.93	4.19
LDL (mg/dl)	107.85	6.46	112.46	6.3	107.5	7.8
BMI (kg/m^2^)	28.99	1.4	30	1.6	29.04	1.2

**Table 3:** Mean ± SEM serum metabolic parameters and BMI in V0, V1, V2 in the two sexes.

	**Females**		**Males**	
	**Mean**	**SEM**	**Mean**	**SEM**
Blood Glucose (mg/dl) V0	81.9	2.9	83.6	3.6
Blood Glucose (mg/dl) V1	81.5	3.9	88	6.1
Blood Glucose (mg/dl) V2	85.3	2.7	82.6	6.5
Triglycerides (mg/dl) V0	177.5	18.9	142.9	26.3
Triglycerides (mg/dl) V1	154.6	21.6	132.6	17.5
Triglycerides (mg/dl) V2	138.2	20.3	146.5	21.9
HDL (mg/dl) V0	51.8	5.1	38.4	2.5
HDL (mg/dl) V1	56.4	6.1	39.6	2.4
HDL (mg/dl) V2	58.5	4.7	39	2.6
LDL (mg/dl) V0	99.9	2.8	113.5	8.8
LDL (mg/dl) V1	120.2	8.5	100.5	7.7
LDL (mg/dl) V2	113.6	9.2	95.3	13.8
BMI (kg/m^2^) V0	28	1.4	31.1	3
BMI (kg/m^2^) V1	28.7	1.6	32.6	3.3
BMI (kg/m^2^) V2	28.4	1.3	31.1	3.1

*No significant differences were observed both between sexes and between follow-up visits*.

**Figure 2:**
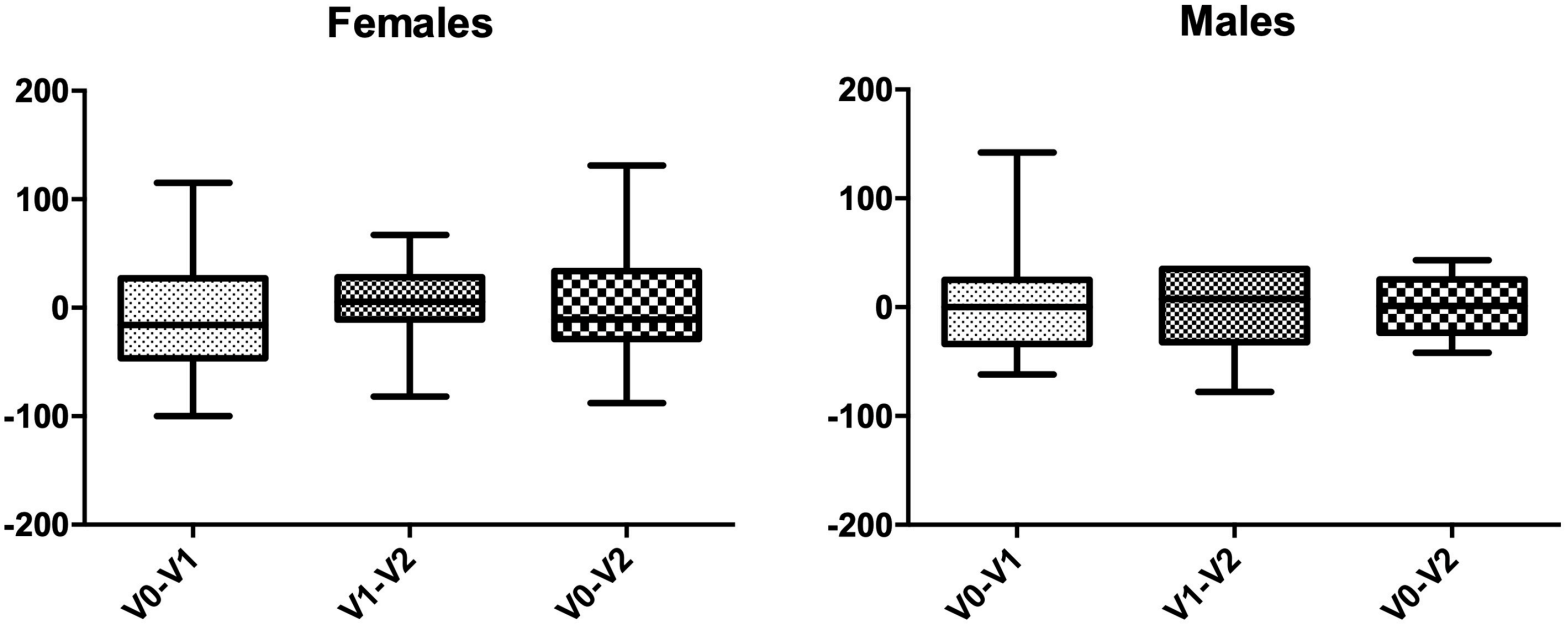
Graphical representation of IGF-1 variations in the two sexes.

**Figure 3:**
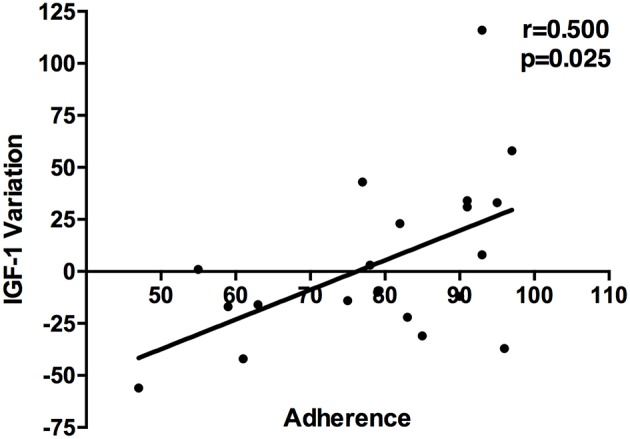
Relationship between adherence and IGF-1 variation throughout the follow up.

As shown in Table 4 there were no differences between mean ± SEM IGF-1 values in the whole population and no differences even when separating males and females (Figure 2). Interestingly enough, a significant correlation between adherence and IGF-1 levels variation during the follow-up was found (Figure 3).

**Table 4:** Mean ± SEM IGF-1 values in V0, V1, V2, and mean ± SEM IGF-1 variations between visits.

**Changes between visits**	**Mean**	**Standard error**	***p***
IGF-1 (ng/ml) V0	137.8	10.20	
V0-V1	−2.61	15.95	NS
IGF-1 (ng/ml) V1	137.46	11.08	
V1-V2	3.92	11.39	NS
IGF-1 (ng/ml) V2	141.04	10.76	
V0-V2	2.80	15.94	NS

Table 5 shows the median adherence rate in patients who were treated only with r-hGH (*n* = 12) and in the ones under replacement therapy for other pituitary deficiencies (*n* = 20).

**Table 5:** Median adherence in subgroups of patients treated with r-hGH alone or with r-hGH plus other hormonal replacement therapy during a 1-year follow up.

**Follow up**	**Population**	**Median adherence rates**
Between V0 and V1	r-h GH alone	88%
	r-h GH plus other^*^	75%
Between V1 and V2	r-h GH alone	83%
	r-h GH plus other^*^	76%
Between V0 and V2	r-h GH alone	84%
	r-h GH plus other^*^	75%

* *These therapies include levothyroxine treatment for secondary hypothyroidism, hydrocortisone treatment for secondary hypoadrenalism, gonadal steroids (testosterone undecanoate or estroprogestinic therapy) for secondary hypogonadism; finally, desmopressin for diabetes insipidus*.

## Discussion

To the best of our knowledge, this is the first study to assess objectively adherence to r-GH treatment in aGHD.

Our results showed, in the group of 32 patients who completed the follow-up, a median adherence rate of 80% throughout V0-V2, lower than the one measured in larger studies on children (11, 14, 15), however still acceptable. Eighty percent, in fact, is the value from which a patient can be considered compliant according to Koledova et al. (14), since it is accompanied by a positive growth outcome in children. Important differences were evidenced between the two genders. The female group can be considered compliant (84% throughout V0-V2), while the males' cannot (71% throughout V0-V2). This might suggest the need for more detailed supervision of the male population. Furthermore, our data confirmed a gradual decrease of compliance during the follow-up both in males and females, as shown, in a longer period, by Koledova et al. (14). Adherence and IGF-1 variation throughout the whole follow-up period were significantly related.

In our study, we did not find significant differences in IGF-1 values during the period of observation as expected, since most of the patients (78%) were already being treated with r-hGH at time of inclusion, with no modification of r-hGH dosage. A similar statement can be also applied to the lack of variation of metabolic parameters, although a trend to lower triglycerides and increased HDL levels in the female subgroup (that also showed better median adherence) was found. However, even in this subgroup, the variability in individual adherence to r-hGH therapy can explain the lack of statistical significance of these variations.

Another important point to be discussed regards the 33 patients who were not included in the final analysis, due to therapy discontinuation or inadequate respect of follow-up schedule. Among the reported motivations above described, some elements led us to hypothesize patients' uncomplete awareness of the importance and benefits of r-hGH replacement therapy. Under this assumption, it is interesting to underline that the adherence rate was higher in the subgroup of patients treated with r-hGH alone than in those with multiple pituitary deficiencies. In the last category, problems related to thyroid and adrenal deficiencies and their life-threatening consequences are most likely considered of greater importance than quality of life and the proven benefits of GH therapy. Moreover, the oral formulation of thyroid and adrenal replacement therapy could be more easily accepted than subcutaneous r-hGH injections. In our opinion, clinical problems related to aGHD are still underestimated, both by physicians and patients, despite a large literature on morbidity and mortality in aGHD patients. Finally, gender related differences in adherence to r-hGH administration could be a field to be further investigated in studies about the psychological profile via specific questionnaires.

A large amount of literature shows beneficial effects of IGF-1 variations during the therapy on metabolic parameters, body weight composition, cardiovascular features (16–18), even if randomized controlled trials are less represented (19). A recent meta-analysis correlates IGF-1 concentrations and efficacy of r-hGH therapy. An increase in IGF-1 >89% has been associated to a significant improvement in heart function and reserve (20), leading to classify patients in responders or non-responders. Before dividing patients in such groups, the adherence to treatment should be evaluated in an objective way.

Easypod^TM^ Connect can represent an adequate tool to individually follow patients and correlate biochemical and clinical improvement to adherence, thus choosing the appropriate dose adjustment. Individualization of replacement therapy has been underlined in the study of Osterziel et al. (21), who explored the administration of different r-hGH schedules in patients with chronic heart failure. Moreover, in the same work, IGF-1 correlated positively with left ventricular ejection fraction and inversely to left ventricular end diastolic and systolic volumes, but only in the group with higher r-hGH administration. IGF-1 response should be evaluated considering both the biological sensitivity to the hormone and the adherence to treatment. The requirement of replacement therapy can vary in individual manner; therefore, a specific management of the single patient is mandatory. This individualization could also allow to avoid waste of unutilized drug, with pharmaco-economic advantage. Awareness of these aspects is a milestone of patient-physician relationship.

There are several potential restrictions to consider in the present study. Firstly, the number of subjects is small, so its statistical power is limited; our findings will need to be confirmed in a larger population. Secondly, follow-up time is short (considering the chronical use of r-hGH in such patients) and longer follow-up is necessary to certify our results. Moreover, this prospective study and its power analysis cannot draw a cause-effect conclusion about adherence/IGF-1 in aGHD patients. Finally, the metabolic profile of individual patients should be added to IGF-1 determination in evaluating the biological response. Further studies are certainly needed to confirm these data, granting a future achievement of a more personalized management of r-hGH replacement therapy.

## Author Contributions

AM and EV were involved in conception and design of the study and wrote the manuscript. EV, CB, AP, and AB were involved in acquisition and analysis of data. All authors were involved in critical discussion and approved the final manuscript.

### Conflict of Interest Statement

The authors declare that the research was conducted in the absence of any commercial or financial relationships that could be construed as a potential conflict of interest.
